# Effects of Exercise-Based Pulmonary Rehabilitation in Patients with Long COVID: A Systematic Review and Meta-Analysis

**DOI:** 10.3390/arm94020025

**Published:** 2026-04-10

**Authors:** Janne Marques Silveira, Ana Paula Midori Nakaishi, Marcos Gontijo da Silva, Daniele Oliveira dos Santos, Ada Clarice Gastaldi

**Affiliations:** 1 Graduate Program in Rehabilitation and Functional Performance, Ribeirão Preto Medical School (FMRP-USP), University of São Paulo—USP, Ribeirão Preto 14049-900, São Paulo, Brazil; jannems@usp.br (J.M.S.); daniolivfisio@gmail.com (D.O.d.S.); 2 Medicine (Undergraduate Program), University of Gurupi (UNIRG), Gurupi 77403-080, Tocantins, Brazil; 3 Physiotherapy Course, Ribeirão Preto Medical School (FMRP-USP), University of São Paulo—USP, Ribeirão Preto 14049-900, São Paulo, Brazil; anapaulanakaishi@gmail.com; 4 Graduate Program in Biotechnology, Federal University of Tocantins, Gurupi 77402-970, Tocantins, Brazil; gontijobio@uft.edu.br

**Keywords:** post-acute COVID-19 syndrome, rehabilitation, systematic review, physical therapy

## Abstract

**Highlights:**

**What are the main findings?**

- In-person pulmonary rehabilitation significantly improves functional exercise capacity in Long COVID, with a clinically meaningful + 53.7 m increase in 6MWT.
- Functional gains were consistent across randomized and observational studies, accompanied by improvements in dyspnea and quality of life, independent of spirometric changes.

**What are the implications of the main findings?**

- Supervised exercise-based pulmonary rehabilitation is associated with improvements in functional recovery in Long COVID.
- Benefits were observed across different study designs, supporting the role of supervised rehabilitation in the multidisciplinary management of Long COVID.

**Abstract:**

**Background/Objective**: A substantial proportion of infected individuals develop persistent symptoms after the acute phase of COVID-19, regardless of initial disease severity. Long COVID (LC) remains a public health challenge characterized by impaired functional exercise capacity (FEC) and quality of life (QoL). We systematically synthesized evidence on the effects of in-person outpatient pulmonary rehabilitation (OPR) with individualized and supervised exercise in adults with LC. **Methods**: Following PROSPERO (CRD42023389365), this study reviewed randomized controlled trials (RCTs) and observational cohort studies (OCSs) published between November 2019 and January 2026 in MEDLINE/PubMed, Web of Science, PEDro, and EMBASE. **Results**: Fifteen studies (*n* = 803) were included. OPR improved FEC (6MWT; MD: 53.72 m, 95% CI 43.69–63.75) and 30″SST (MD: 4.68, 95% CI 3.59–5.77) and reduced exertional dyspnea. RCTs showed benefits in physical (MD: 8.04, 95% CI 3.02–13.05) and mental QoL (MD: 6.60, 95% CI 2.01–11.18) and dyspnea impact, with inconsistent PF findings. Fatigue showed a trend toward improvement but was measured using heterogeneous patient-reported tools in RCTs and OCSs. **Conclusions**: Supervised PR improves FEC, QoL, and dyspnea in individuals with LC. In patients with fatigue/PEM, systematic assessment and continuous symptom monitoring are essential. High-quality controlled studies are needed to strengthen evidence and clinical guide.

## 1. Introduction

Long COVID (LC) is considered a significant post-pandemic health problem that manifests itself regardless of the severity of COVID-19 [1,2]. It is characterized by long-lasting, heterogeneous, and multisystemic symptoms. Prevalence estimates remain high, ranging from 36% [3], 45% [4] to 57% [5]. Moreover, longitudinal data indicate persistence and even increase over time, from 35% at the 12-month to 46% at the 12- to 24-month follow-up after the acute phase of COVID-19 [3].

The clinical and functional implications of LC [6] reduce physical performance and exercise tolerance [7,8]. Such limitations are related to a greater sensation of dyspnea at rest, which worsens during exercise, negatively impacting activities of daily living (ADL) [9] and QoL [10]. In addition, they compromise productivity and work capacity, overburdening health systems worldwide [11].

The course of spontaneous recovery from COVID-19 varies, with improvement most likely occurring within 6 months. However, a substantial proportion of people tend not to recover fully and require specific interventions aimed at functional improvement and symptom management [12].

Classically, PR is a well-established strategy in the management of a variety of chronic respiratory diseases [13]. Patients with LC may benefit from physical therapy programs that utilize various exercise modalities combined with in-person, remote, or hybrid therapeutic interventions. However, because these programs involve multimodal interventions, the specific effects of exercise alone have not been clearly established, particularly given the clinical heterogeneity of LC and the potential risk of adverse events [14].

In this context, given that the effects of protocols focused primarily on physical exercise for patients with LC have not yet been clearly established in the literature [15], it is necessary to evaluate the effects of exercise-based physical therapy. This modality was chosen because it is a strategy that does not require complex infrastructure or sophisticated technologies and is easy to implement at different levels of healthcare [16].

Given this, this systematic review aimed to identify the best evidence from the literature on the impact of in-person and supervised outpatient pulmonary rehabilitation, with physical exercise as the main strategy, on functional exercise capacity, pulmonary function, and dyspnea in patients with LC.

## 2. Materials and Methods

### 2.1. Register

This review adhered to the PRISMA (Preferred Reporting Items for Systematic Review and Meta-Analyses) guidelines [17] and was registered for access in the International Prospective Registry of Systematic Reviews (PROSPERO) under protocol number n° CRD42023389365 for access. This study was partially funded by the Coordination for the Improvement of Higher Education Personnel—Brazil (CAPES) and no conflicts of interest affected this review.

### 2.2. Eligibility Criteria

Randomized clinical trial (RCTs) and observational cohort studies (OCS) were included.

### 2.3. Participants

Participants were adults aged 18 years and older diagnosed with LC, which is defined as persistent symptoms continuing from 12 weeks after SARS-CoV-2 infection [1], regardless of the clinical form of COVID-19 in the acute phase.

### 2.4. Intervention

Outpatient pulmonary rehabilitation (OPR) lasts at least 4 weeks, with supervised physical training as a unique and essential component. Interventions in RCTs were compared to any control.

### 2.5. Outcomes

Primary outcomes included FEC as assessed by the 6-Minute Walk Test (6MWT) or Sit-to-Stand Test (SST) and QoL. Secondary outcomes included PF assessed by spirometry, dyspnea at rest measured by the modified Medical Research Council (mMRC) scale, and dyspnea during exercise assessed by the adapted Borg Scale.

### 2.6. Exclusion Criteria

Studies not available in English, editorials, letters, congress and conference abstracts, case reports and series, preprints, pilot studies and studies describing protocols, and those not meeting the eligibility criteria were excluded.

### 2.7. Search, Selection and Data Extraction Process

Searches used MeSH terms (Medical Subject Headings) and were customized for each database, Pubmed, Web of Science, Physiotherapy Evidence Database (PEDro) and EMBASE via Elsevier, for studies published between November 2019 and January 2026.

The concepts “COVID-19” AND “Rehabilitation” OR “Exercise Therapy” AND “Walk Test” OR “Sit and Stand Test” OR “Quality of life” OR “Respiratory Function Tests” OR “Dyspnea” were used in the search. The concept map and search strategy can be viewed in Supplementary Materials S1 and S2.

Deduplication was performed using Rayyan^®^ software [18]. Two reviewers (JMS and APMN) independently conducted selection, data extraction and risk of bias analysis with discrepancies resolved by a third reviewer (DOS). Authors were contacted for unavailable data in included studies. Most of our requests were successful.

A standardized form was used with the following information on the articles included: year of publication, authors, type of study, title, characteristics of the participants (age and clinical classification of COVID-19 in the acute phase), number of participants before and after the intervention, intervention details (duration, parameters and follow-up period) and primary and secondary outcomes (accessed in the Eligibility Criteria/Outcomes subsection). When multiple assessments occurred during follow-up, data from the end of the intervention protocol were included. For RCTs, we also collected data from the control group for comparison with the intervention group with OPR.

### 2.8. Assessing the Risk of Bias

The National Heart, Lung and Blood Institute (NHLBI) [19] criteria specific to RCTs and OCSs were used. For the RCTs, criteria and questions were: randomization (1), allocation (2, 3), blinding (4, 5), characteristics of the groups at baseline (6), dropout (7, 8), adherence (9), deviation from the planned intervention (10), validity of the outcome measures (11), power of the test for sample calculation (12), prespecified outcome (13), intention-to-treat analysis (14). Responses were categorical: “yes”, “no” and “not reported”. For OCSs, criteria and questions were: clarity research question and objective (1), population recruitment and standardization (2,3), uniform group eligibility criteria (4), sample size justification (5), exposure assessment prior to outcome measurement (6), adequate time to observe effects (7), various exposure levels (8), definition of exposure measure/independent variable measures (9), repeated exposure assessment (10), outcome measures (11), blinding of outcome assessors (12), follow-up rate (13), statistical analysis (14). Responses were categorical: “yes”, “no” and “not reported”. The overall risk was categorized according to the number of “yes” responses: 1–8 (high risk), 9–12 (unclear risk), and 13–14 (low risk), in accordance with similar systematic review [20].

The response synthesis/global bias classification for each study indicated low, unclear and high risk of bias, respectively. Disagreements between independent reviewers were resolved by a third reviewer.

The graphical tool Canva was used to generate individual and global risk of bias figures for the included studies. Methodological quality of this systematic review was assessed using the Measurement Tool to Assess Systematic Reviews 2 (AMSTAR 2).

### 2.9. Synthesis and Analysis

Data originally reported as median and interquartile range were converted to mean and standard deviation for the meta-analyses, according to statistical methods described in the literature [21]. When presented in figures, data were extracted using WebPlotDigitizer [22]. If meta-analysis was not possible, a descriptive synthesis was carried out.

Quantitative analyses of continuous variables were grouped as mean difference (MD) with 95% confidence interval (95% CI) at pre- and post-intervention. The meta-analyses were conducted separately according to study design, with independent analyses of observational studies (OCSs) and randomized clinical trials (RCTs), with the aim of reducing methodological heterogeneity and increasing the robustness of the estimates. Interventions in RCTs were compared to the control group.

In the meta-analyses, the results were expressed as MD 95% CI for the outcomes of FEC assessed by the 6MWT and 30″SST, 5 dimensions of QoL (EQ 5D) were assessed using the visual analog scale (EQ/VAS), and dyspnea was assessed by the Borg scale and in the OCS. In the RCTs, meta-analyses were performed on QoL assessed by the Short Form12 (SF-12), physical (QoL/PD) and mental (Qol/MD) domains, pulmonary function parameters (FVC, FEV_1_, FEV_1_/FVC) were measured in accordance with the recommendations of the American Thoracic Society/European Respiratory Society (ATS/ERS), using reference equations from the Global Lung Function Initiative (GLI) [23], and dyspnea was assessed by the mMRC.

A value of 30.5 m was used as the minimally significant clinical difference (MSCD) in the 6MWT [24].

Statistical analysis was performed using R software, version 4.3.1, for Windows 10, meta package version 6.5.0. [25]. In the absence or presence of reduced heterogeneity (I^2^ < 10% and *p* > 0.05), the fixed-effect model was applied using the Mantel–Haenszel method; for greater heterogeneity (I^2^ > 10% and *p* < 0.05), the random effects model was applied using the DerSimonian–Laird method [26]. I^2^ values near 25%, 50% and 75% or more indicated low, moderate and high heterogeneity, respectively [26,27]. In the presence of high heterogeneity, sensitivity analysis was carried out by excluding studies.

### 2.10. Certainty of Evidence

The Grading of Recommendations Assessment, Development, and Evaluation (GRADE) approach was used to assess the certainty of evidence for each outcome. Certainty was considered high for RCTs, which can be downgraded, and low for OCSs, which can be downgraded or upgraded [28].

The certainty of evidence rating for both RCTs and OCSs includes five items that may downgrade the evidence: risk of bias, inconsistency, indirect evidence, imprecision and publication bias [29]. In addition, OCSs include three items that may upgrade the evidence: large treatment effects, dose–response gradient and confounders against treatment.

The level of certainty is high when the results present a low risk of bias, consistency, directness, and precision, suggesting minimal impact from further studies; moderate when new studies are likely to change the effect estimate; low when new studies could significantly affect the estimate; and very low when uncertainty surrounds the effect estimate [30,31].

## 3. Results

Study Selection

**Figure 1:**
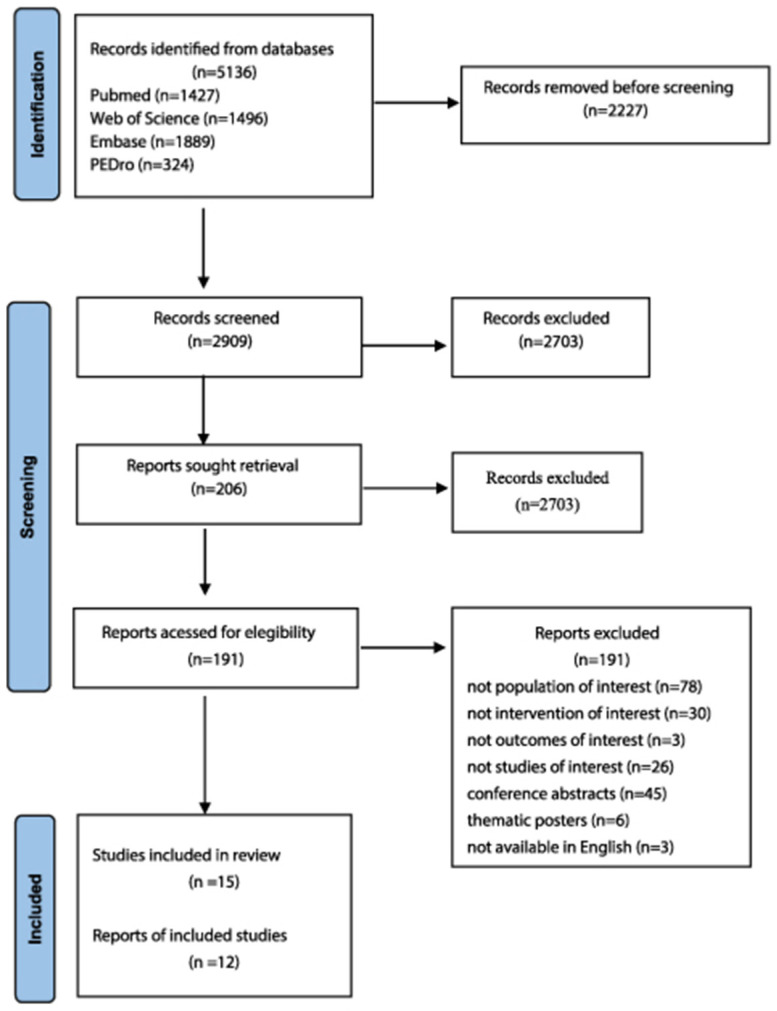
PRISMA flowchart.

The search strategy retrieved 5136 studies, removed 2227 duplicates, and screened 2909 titles and abstracts. Following this stage, 206 studies were read in full, and the reasons for exclusion (*n* = 191) are presented in the PRISMA flowchart. Fifteen studies were finally included (Figure 1). The RCTs included 329 [32,33,34,35,36,37] and the OCSs included 474 [38,39,40,41,42,43,44,45,46] for a total of 803 participants.

### 3.1. Characteristics of the Studies

The characteristics of the included studies can be found in Supplementary Material S3. The results on the effects of the OPR are shown in Table 1 and Table 2. The list of excluded studies is available in Supplementary Material S4.

Of the fifteen included studies, six were RCTs [32,33,34,35,36,37] and nine were OCSs [38,39,40,41,42,43,44,45,46]. All the patients with LC who had persistent symptoms (>3 months after the acute phase) participated in the in-person OPR program with physical exercise as an essential component.

The duration of the protocols was 3 [36], 4 [41,46], 6 [35,38,42,43,44], 7 [40], 8 [33,34,39] 10 [32,37] and 16 [45] weeks in the included studies. The number of weekly sessions varied from 2 [32,37,38,39,40,45], 3 [33,34,35,43,44,46], 5 [36,42] to 6 [41].

FEC was assessed in nine studies with 6MWT [35,36,39,41,42,43,44,45,46], four with 30″SST [39,43,44,45], one with a 1-min Sit-to-Stand Test (1′SST) [42] and one with a five-repetition Sit-to-Stand Test (5 × SST) [33]. QoL was assessed with SF-12 (physical and mental domains) in three studies [33,34,41] and with EQ-5D-5L in four studies [32,37,38,40]. PF was assessed in three studies [33,35,36]. Dyspnea was assessed with mMRC in six studies [33,34,35,43,44,46]. Three studies assessed dyspnea using the Borg scale [35,43,44], and one study assessed dyspnea using the Dyspnea-12 (D-12) [32].

Post-intervention QoL did not improve in two RCTs, one evaluated by EQ 5D (EQ Index and EQ/VAS) [32] and the other in the physical and mental domains of SF-12 [34]. Two OCSs showed no improvement in this same outcome by EQ/VAS [38] and in the mental domain of SF-36 [46]. PF and its variables (FVC, FEV1, and FEV_1_/FVC) did not change after the intervention in two RCTs [33,35], with significant improvement reported in only one RCT [36]. As for dyspnea, two RCTs did not show improvement in this outcome in the intervention group when assessed by mMRC [33] D-12 [32].

### 3.2. Risk of Bias

**Figure 2:**
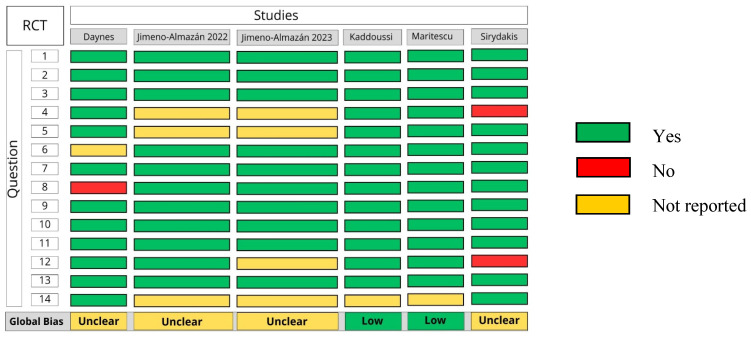
Risk of bias using the specific National Heart, Lung and Blood Institute (NHLBI) for randomized clinical trials [32,33,34,35,36,37].

**Figure 3:**
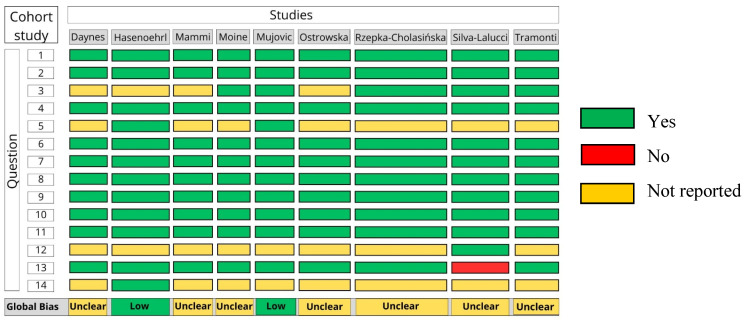
Risk of bias using the specific National Heart, Lung and Blood Institute (NHLBI) for observational cohort studies [38,39,40,41,42,43,44,45,46].

Regarding the overall risk of bias among the included RCTs, four [32,33,34,37] presented an unclear risk of bias, and two [35,36] presented a low risk, related to the absence of blinding of evaluators, incomplete description of losses, lack of prior sample calculation, and no intention-to-treat (ITT) analysis (Figure 2). In the OCSs, seven studies presented an uncertain risk [38,40,41,43,44,45,46] and two presented a low risk [40,42], predominantly associated with the absence of adequate control of confounding factors and the absence of adjusted analyses (Figure 3).

Criteria assessed and question number:

Randomization: 1

Allocation: 2, 3

Blinding: 4, 5

Group characteristics at baseline: 6

Dropout: 7, 8

Adherence: 9

Deviation from planned intervention: 10

Validity of outcome measures: 11

Power of the test for sample calculation: 12

Prespecified outcomes: 13

Intention-to-treat analysis: 14

Criteria assessed and question number:

Research question and goal defined: 1

Study population: 2, 3

Recruitment of groups from the same population with uniform eligibility criteria: 4

Sample size justification: 5

Exposure assessed prior to outcome measurement: 6

Sufficient time frame to see an effect: 7

Different levels of the exposure of interest: 8

Definition of exposure measures/independent variable: 9

Repeated evaluation of exposure measures: 10

Outcome measures: 11

Blinding of outcome assessors: 12

Follow-up rate: 13

Statistical analysis: 14

### 3.3. Publication Bias

The funnel plot was constructed for exploratory analysis of publication bias in the meta-analysis of the FEC (6MWT) outcome.

### 3.4. Effectiveness of OPR on FCE, QoL, PF, and Dyspnea

In the quantitative syntheses evaluating FEC outcomes (6MWT and 30″SST), the original pre- or post-intervention data stratified by COVID-19 severity in two of the included OCS [39,45] were pooled using a weighted mean.

**Figure 4:**
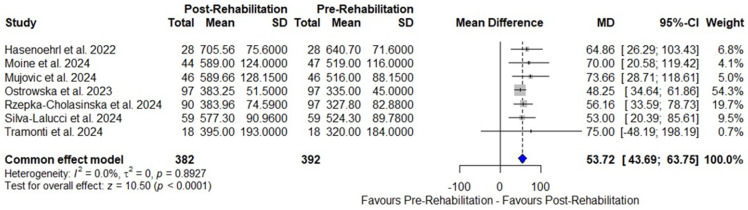
Benefit of OPR using the 6MWT (meters) in OCSs [39,41,42,43,44,45,46].

**Figure 5:**
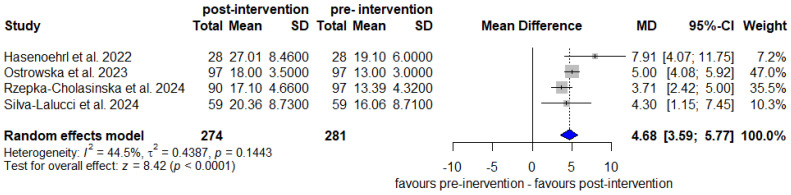
Benefit of OPR using the 30″SST (repetitions) in OCSs [39,43,44,45].

Meta-analysis of seven OCSs [39,41,42,43,44,45,46] demonstrated an improvement in FEC during exercise using the 6MWT (MD: 53.72 m, 95% CI 43.69–63.75, I^2^ = 0%, *p* = 0.89) (Figure 4). Another meta-analysis with four OCSs [39,43,44,45] also demonstrated an improvement assessed by the 30″SST (MD: 4.68 repetitions, 95% CI 3.59–5.77, I^2^ = 44.5%, *p* = 0.14) (Figure 5), both with very low certainty of evidence. The sensitivity analysis of the 30″SST by removing one study [39] that included patients under the age of 50 from the other three studies [43,44,45] that included patients over the age of 50 identified a reduction in heterogeneity to 22.3% (MD: 4.45, 95% CI 3.40–5.49, I^2^ = 22.3%, *p* = 0.27) (Supplementary Material S5). In two individual studies, one RCT [33] and one OCS [42] showed a significant improvement in FEC by the 5 × SST and 1′SST tests, respectively.

**Figure 6:**
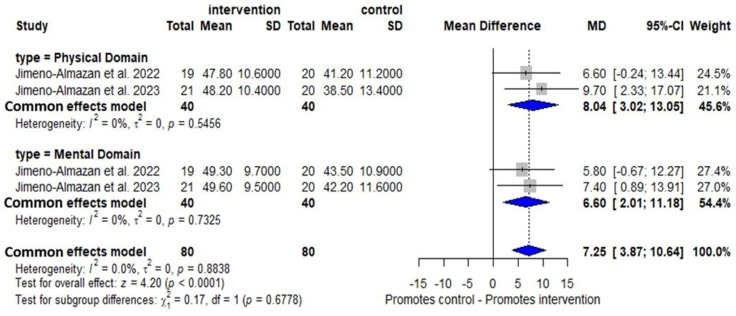
Benefit of OPR on QoL/physical and mental domains in RCTs [33,34].

Two RCTs [33,34] demonstrated improved QoL after intervention in the physical (MD: 8.04, 95% CI 3.02–13.05, I^2^ = 0%, *p* = 0.54) and mental (MD: 6.60, 95% CI 2.01–11.18, I^2^ = 0%, *p* = 0.73) domains compared with the control group, both with low certainty of evidence (Figure 6). Two OCSs [38,40] showed an improvement in QoL EQ/VAS (MD: 14.16, 95% CI 3.23–25.09, I^2^ = 70%, *p* = 0.07) with low certainty evidence (Supplementary Material S6). There was also a significant improvement in this outcome using the EQ/VAS after the intervention with physical exercise in one RCT [37] and in one OCS [46] in the physical domain of the SF-36.

**Figure 7:**
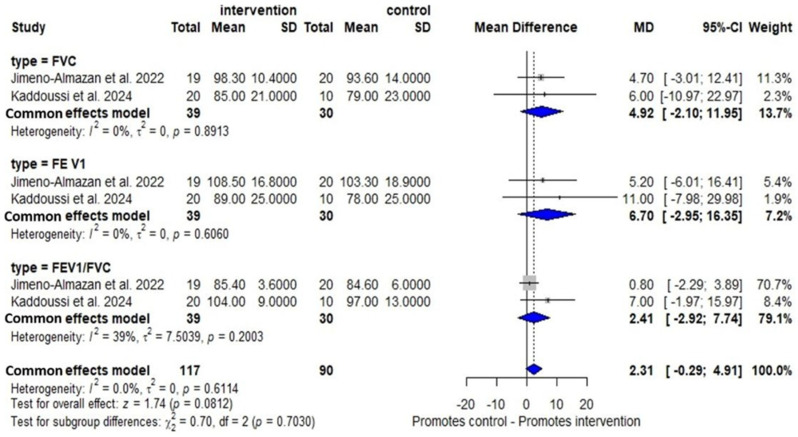
Results of OPR on PF in RCTs [33,35].

Two RCTs [33,35] showed that there were no changes in the variables of the PF in FVC (MD: 4.92; 95% CI −2.10–11.95, I^2^ = 0%, *p* = 0.89), in FEV_1_ (MD: 6.70; 95% CI −2.95–16.35, I^2^ = 0%, *p* = 0.60) and in FEV_1_/FVC (MD: 2.41, 95% CI −2.92–7.74, I^2^ = 39%, *p* = 0.20) post-intervention compared with the control group (Figure 7). The certainty of the evidence was low for all three PF parameters assessed.

**Figure 8:**
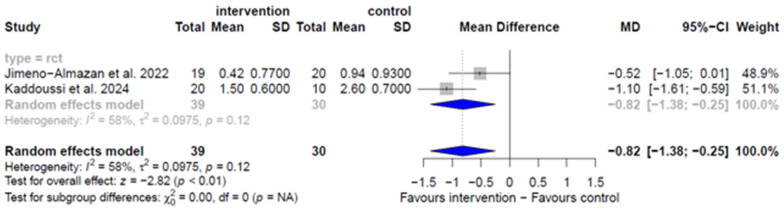
Benefits of OPR for dyspnea by mMRC in RCTs [33,35].

Meta-analysis of the two RCTs [33,35] showed a reduction in dyspnea assessed by the mMRC in the impact of dyspnea on ADLs (MD: −0.82, 95% CI −1.38–−0.25, I^2^ = 58%, *p* = 0.12) (Figure 8) in post- intervention compared with the control group with very low certainty of evidence. Another meta-analysis with two OCSs [43,44] showed a reduction in exertional dyspnea assessed by the Borg scale (MD: −1.14, 95% CI −1.51–−0.77, I^2^ = 46%, *p* = 0.18) after the intervention with very low certainty of evidence (Supplementary Material S7). Individual studies showed a significant reduction in dyspnea assessed by the mMRC [34,43,44,46] and Borg scale [35].

The certainty of the evidence for all outcomes is available in Supplementary Material S8.

The funnel plot assessing publication bias in the meta-analysis of FEC using the 6MWT is available in Supplementary Material S9. The observed asymmetry (*p* = 0.01) should be interpreted with caution given the small number of studies included in the meta-analysis (*n* < 10).

## 4. Discussion

Exercise-based pulmonary rehabilitation promoted improvements in FEC, QoL, and reductions in dyspnea related to ADL and exertion, without consistent evidence of improvement in PF. Several pulmonary and extra-pulmonary factors limit exercise capacity in patients with LC, contributing to an increased perception of dyspnea. This symptom may be present at rest but typically worsens during physical effort, leading to functional limitation and impaired QoL [10,47,48].

In the included studies, the duration of PR protocols ranged from 3 to 16 weeks. Evidence from chronic respiratory disease suggests that longer protocols, ranging from 8 to 12 weeks, were associated with greater potential gains [49]. Protocols with durations lasting 8 [33,34,39] and 16 weeks [45] in the present review are consistent with this perspective. Nevertheless, the benefits of PR were also identified in shorter protocols lasting three [36], four [41,46], six [35,38,42,43,44] and seven [40] weeks.

Regarding FEC, OCS meta-analyses demonstrated significant post-intervention improvement. The meta-analysis of seven studies [39,41,42,43,44,45,46] demonstrated a mean increase of 53.72 m in 6MWT, exceeding the 30.5 m MSCD, which was adopted from other chronic lung diseases [24] given the absence of a defined value for LC. The absence of heterogeneity (I^2^ = 0) should be interpreted with caution, as the small number of included studies (*n* = 7) reduces the statistical power to detect true heterogeneity. Additionally, clinical differences related to the severity of COVID-19 in the acute phase and participant characteristics, as well as relevant methodological differences regarding the varying durations of the intervention protocols, should be considered. In three of the included studies [43,44,46], patients performed below 350 m, indicative of a worse prognosis in chronic lung diseases [50,51,52]. Moreover, previous evidence indicates [53] that patients with LC, even with preserved PF, performed below the 50th percentile of reference values for healthy individuals in the 6MWT. Another study [54] showed that patients with LC after a mild clinical case of COVID-19 walked less than 80% of what was predicted. Given the similarity of clinical manifestations between LC and other chronic lung diseases.

Taken together, these findings reinforce the clinical relevance of the observed gains. The meta-analysis of the 30″SST [39,43,44,45] demonstrated a mean increase of 4.68 repetitions post-intervention. Moderate inconsistency (I^2^ = 44.5%) was reduced to low (I^2^ = 22.3%) after excluding one study [39] whose participants were younger than 50 years, differing from those in the other included studies (>50 years), suggesting that age may influence performance in submaximal functional tests, reinforcing the need to individualize exercise prescription in PR programs [55].

Studies not eligible for the meta-analyses due to presenting methodological designs different from those included [35,36] or for using different instruments to assess the same outcome, such as the 5 × SST [33] and the 1′SST [42], demonstrated improvement after the intervention.

With respect to QoL, the RCTs’ meta-analysis [33,34] demonstrated an improvement in both physical (I^2^ = 0%) and mental domains (I^2^ = 0%) of the SF-12 in the intervention group. The lack of heterogeneity observed in both domains, although it suggests a high degree of clinical homogeneity (age, clinical profile, and disease severity) and methodological homogeneity (8-week protocol), should be interpreted with caution, given that only two studies were included; low statistical power may mask variability and overestimate the consistency of the findings. As the OPR program included in this review had physical exercise as an essential component, despite any structured psychotherapeutic intervention, it was also possible to observe the positive impact on the mental domain, which suggests that there is a potential indirect psychological benefit associated with physical training. A previous systematic review [56] reported that in-person protocols seem to be superior to remote protocols for improving the mental component in patients with LC. On the other hand, one OCS [46] that used the SF-36 identified improvement only in the physical domain, suggesting that the mental domain may exhibit different sensitivity depending on the instrument used, particularly when exercise-based intervention is not associated with a structured psychological intervention.

With respect to quality of life, the meta-analysis of two OCSs [38,40] demonstrated improvement in QoL as measured by the EQ/VAS, with a mean increase of 14.16 points. The substantial inconsistency (I^2^ = 70%) observed may be attributed to differences in the age of participants, intervention duration, exercise modality, and training volume across the studies. In contrast, a recent study [32] did not find improvement in QoL assessed by the EQI or EQ/VAS post-intervention compared with the control group. The authors attributed this finding to the heterogeneous symptoms of LC and to a possible ceiling effect reached by some participants.

The meta-analysis of two RCTs [33,35] did not demonstrate the effects of the intervention on PF (I^2^ = 0). A possible explanation is that patients presented baseline spirometric values within the normal range, thereby limiting the potential to detect measurable improvement. Although recent evidence [16] associated exercise with reduced airway inflammation, its effects on PF in patients with post-COVID-19 pulmonary sequelae remain inconclusive [57], and the evidence is still limited [16]. Despite differences in clinical characteristics, participant age, and protocol duration, the small number of studies (*n* = 2) limits the statistical power to detect true heterogeneity.

Regarding dyspnea, the meta-analysis with two RCTs [33,35] demonstrated that exercise-based rehabilitation reduces the impact of severe dyspnea (mMRC ≥ 2) on ADLs. Moderated inconsistency (I^2^ = 58%) may be attributed to differences among the studies regarding the severity of COVID-19 in the acute phase (mild vs. mild to severe), age (<50 vs. >50 years), and study duration (8 vs. 6 weeks). An additional RCT [34] not included in the meta-analysis reported similar findings, demonstrating a significant reduction in the proportion of patients with severe dyspnea (mMRC ≥ 2). In contrast, one RCT [32] did not identify a significant improvement in dyspnea in the intervention group when assessed using the D-12 instrument. This tool captures the affective dimension of dyspnea, which may have been less responsive to an exercise-based intervention without a structured psychological approach. One OCS [41] identified a significant reduction in dyspnea as measured by the mMRC.

With respect to the dyspnea post-intervention, reduction was assessed by the Borg scale and was observed in the meta-analysis of two OCSs [43,44] (I^2^ = 46%). Although the studies were similar in terms of duration (6 weeks) and age (≥60 years), the differences are likely due to the clinical characteristics of the participants, including patients with severe COVID-19 in only one study [43], as well as the limited number of studies (*n* = 2). One RCT [35] also demonstrated a significant reduction in exertional dyspnea in the intervention group compared with the control group.

### 4.1. Long COVID and Fatigue

Fatigue was not originally included in the PICO framework, although it emerged as a relevant outcome in this review. Among the more than 200 symptoms described in LC [58], fatigue is the most frequently reported and one of the most disabling symptoms [59].

Current recommendations emphasize the use of appropriate instruments to define fatigue, assess its functional impact, and guide clinical management [60,61]. In the included studies, fatigue was assessed by different subjective self-reporting instruments [32,33,34,38,39,41,42,43,44], and reduction following exercise-based rehabilitation was reported in two RCTs [33,34] and in six OCSs [38,39,41,42,43,44]. Two additional studies [37,40] showed a reduction in fatigue after intervention based only on patient reports, without standardized measurement instruments.

Most scales used measured the intensity of fatigue and its functional impact but did not apply the diagnostic criteria. Only two of the included studies [32,33] used the DePaul Symptom Questionnaire (DSQ) [62], which is an instrument for screening and diagnostic confirmation of chronic fatigue syndrome (CFS). In addition to assessing the frequency and intensity of fatigue, it evaluates criteria related to post-exertional malaise (PEM), considered a central feature of this symptom [63] and an adverse event related to exercise. One of the studies used the DSQ to screen for fatigue [33], whereas another [32] applied it to identify clinical improvements in this symptom after intervention compared to the control group. Three studies [32,37,41] reported adverse events. In two of them [32,37], these were unrelated to fatigue, and only one study [41] identified a case of PEM.

The assessment of fatigue relied on a predominantly heterogeneous and subjective self-reporting instrument, which preludes quantitative synthesis and limits the interpretation of the results. However, findings regarding fatigue should be interpreted with caution, as their assessment in the included studies was based on heterogeneous self-reporting instruments measuring perceived functional outcomes, without the systematic application of formal diagnostic criteria for chronic fatigue syndrome/post-exertional malaise (CFS/PEM). This limitation compromises adequate clinical stratification and hinders the identification of the subgroup with fatigue associated with PEM, a condition that requires specific therapeutic approaches.

### 4.2. Clinical Implications

The pulmonary rehabilitation (PR) protocol, which is primarily exercise-based, is well tolerated by patients and leads to clinically significant improvements in exercise capacity (EC), quality of life (QoL), and a reduction in dyspnea, underscoring the importance of exercise in structured PR protocols for the management of Long COVID [64]. Interventions based primarily on exercise should be considered a central component and integrated into the care of patients with LC. However, their implementation requires rigorous clinical stratification, individualized prescribing, and continuous monitoring, combined with professional training. In the context of public policy, exercise as the primary strategy in physical rehabilitation represents a low-cost approach with minimal infrastructure and technology requirements [16], thereby expanding the population’s access to pulmonary rehabilitation.

The in-person, supervised, and individualized delivery model of pulmonary rehabilitation (PR) is strategic, as it allows rigorous clinical monitoring of exercise responses, precise adjustment of intensity, and personalized prescription, which is particularly important for patients at risk of symptom exacerbation, such as those in the fatigue/post-exertional malaise (PEM) subgroup. On the other hand, alternative models, including remote strategies, may impose barriers related to the environment and the availability of technological resources [65], in addition to increasing the risk of PEM worsening due to the lack of in-person monitoring, which may compromise adherence to PR.

Regarding fatigue, particularly in the fatigue/PEM subgroup, although rehabilitation is indicated, interventions should be adapted and individualized to ensure greater patient safety, optimize functional outcomes, and prevent symptom exacerbation. Additionally, the findings suggest the need to standardize the assessment of patients with Long COVID using specific and validated instruments, combined with continuous and routine monitoring to minimize the risk of adverse events, thereby enhancing the safety of interventions.

### 4.3. Strengths and Limitations

The search strategy was updated to January 2026, ensuring the inclusion of the most recent evidence. Restricting inclusion to individuals ≥3 months after the acute phase of COVID-19 enhanced clinical homogeneity and alignment with current definitions of LC. Additionally, the interventions included traditional, in-person, and individualized PR programs, based on established models for chronic lung diseases, supporting feasibility and external validity. The delivery model reinforces clinical safety in the face of potential adverse events, while allowing individualized adjustments to address the heterogeneity of LC manifestations, particularly PEM.

It was not possible to determine the influence of the severity of COVID-19 in the acute phase on the effects of PR. Although the protocols were in-person and individualized, applied to patients with LC (≥3 months) after the acute phase, most studies did not consider the clinical diagnosis of fatigue and did not monitor exercise-related adverse events. These findings are consistent with previous Cochrane reviews [66,67] which report the potential benefits of PR but highlight low certainty of evidence and insufficient consideration of fatigue and PEM management.

The included studies exhibited methodological limitations, with a high or unclear risk of bias, mainly due to the absence of sample size calculation, small sample sizes, limited control of confounding variables, and lack of blinding, as well as the limited inclusion of RCTs. The risk of bias assessment using NHLBI instruments favors internal comparability among the studies included in this review. However, other tools such as RoB 2 and ROBINS-I could have been used, as they provide a more detailed framework for assessing bias domains aligned with outcomes.

The certainty of the evidence for the outcomes of interest was rated as low or very low, reinforcing the need for caution in interpreting the findings, generalizing the results, and drawing definitive conclusions.

Future trials should adopt higher methodological standards, incorporate structured diagnostic tools for CFS/PEM, and systematically report adverse events to enhance the safety and interpretability of exercise-based PR in this subgroup. Additionally, given the higher prevalence and functional impact of LC among middle-aged adults and women, future research should prioritize stratified analyses to improve clinical applicability [68,69]. As future perspectives, more robust studies with better methodological quality that incorporate the systematic diagnosis of CFS/PEM are needed to ensure greater safety in PR focused on exercise for this subgroup of patients.

### 4.4. Contributions

This systematic review synthesizes evidence on exercise-based pulmonary rehabilitation for Long COVID, providing a better understanding of the specific effects of this intervention in a context often characterized by multimodal approaches. The results demonstrate improvements in exercise capacity, quality of life, and dyspnea, while also highlighting significant gaps, such as the absence of consistent effects on lung function and limitations in the assessment of fatigue, particularly due to the lack of formal diagnostic criteria for post-exertional malaise (PEM). From a clinical perspective, the findings reinforce the importance of supervised protocols, with individualized prescriptions and continuous monitoring, particularly to reduce the risk of symptom exacerbation in patients with fatigue/PEM. Furthermore, they highlight exercise as a safe, low-cost strategy requiring minimal infrastructure, favoring its broad applicability across different levels of healthcare. Finally, this study guides future research regarding the need for greater methodological rigor, standardization of protocols and outcomes, as well as the systematization of assessment and continuous monitoring, especially in subgroups at higher risk of post-exercise exacerbation.

## 5. Conclusions

In-person, supervised PR with structured exercise improves exercise capacity, QoL, and dyspnea in individuals with LC. In patients with fatigue/PEM, systematic assessment and continuous symptom monitoring are essential to prevent the risk of symptomatic exacerbation and ensure the safety of interventions. Future high-quality trials incorporating standardized diagnostic criteria and adverse-event reporting are needed to optimize patient selection and intervention safety.

## Figures and Tables

**Table 1:** Outcomes of Randomized Clinical Trials (RCTs).

Author/Year	Daynes et al./2025 [32]	Jimeno-Almazán et al./2022 [33]	Jimeno-Almazán et al./2023 [34]	Kaddoussi et al./2024 [35]
Risk of bias	Unclear	Unclear	Unclear	Low
Participants	IG (*n* = 56) vs. CG (*n* = 62)	IG (*n* = 19) vs. CG (*n* = 20)	IG (*n* = 21) vs. CG (*n* = 20)	IG (*n* = 20) vs. CG (*n* = 10)
Intervention	IG—OPR supervised, personalized, in-person (2×/week aerobic and resistance exercises), combined with 3 home-based sessions of strength and resistance and self-management strategies for 8 consecutive weeks. CG—usual care.	IG—OPR supervised with multicomponent, personalized exercises, 3×/week (2× moderate intensity aerobic exercises and 1× light intensity continuous training) for 8 consecutive weeks. CG—unsupervised, which followed the WHO guidelines for rehabilitation after COVID-19.	IG—OPR supervised with multicomponent, personalized exercises, 3×/week (2× moderate intensity aerobic exercises and 1× light intensity continuous training) for 8 consecutive weeks. CG—unsupervised, which followed the WHO guidelines for rehabilitation after COVID-19.	IG—OPR supervised group OPR with aerobic and strength exercises, 3×/week for 6 consecutive weeks. CG—maintenance of the usual level of sedentary physical activity.
Physical Performance (6MWT) (m)				IG (pre × post) vs. GC (pre × post) (349 ± 137 × 517 ± 115) vs. (414 ± 106 × 419 ± 78) (*p* = 0.01)
Physical Performance (5× SST)		IG (pre × post) vs. GC (pre × post) (6.6 ± 2.5 × 5.1 ± 1.2) vs. (8.3 ± 3.5 × 6.6 ± 1.5) (*p* = 0.009)		
SF-12 QoL/PD		IG (pre × post) vs. CG (pre × post) (35.7 ± 11.6 × 47.8 ± 10.6) vs. (37.2 ± 11.0 × 41.2 ± 11.2) (*p* = 0.024)	IG (pre × post) vs. CG (pre × post) (35.2 ± 11.6 × 48.2 ± 10.4) vs. (36.5 ± 11.7 × 38.5 ± 13.4) (*p* < 0.001)	
SF-12 QoL/MD		IG (pre × post) vs. CG (pre × post) (46.1 ± 12.2 × 49.3 ± 9.7) vs. (39.6 ± 11.5 × 43.5 ± 10.9) (*p* = 0.444)	IG (pre × post) vs. CG (pre × post) (46.3 ± 11.9 × 49.6 ± 9.5) vs. (39.5 ± 12.1 × 42.2 ± 11.6) (*p* > 0.05)	
EQ 5D QoL	IG (pre × post) vs. CG (pre × post) in MD/IQR VAS [57.75 (52.44–63.05) × 62.40 (57.14–67.65)] vs. [60.84 (55.59–66.09) × [65.61(60.75–70.48)] (*p* > 0.05) EQI [0.58 (0.53–0.63) × 0.61 (0.56–0.66)] vs. [0.59 (0.52–0.66) × 0.64 (0.59–0.69)] (*p* > 0.05)			
PF		IG (pre × post) vs. CG (pre × post) FVC (%) (97.2 ± 13.7 × 98.3 ± 10.4) vs. (94.1 ± 13.8 × 93.6 ± 14.0) (*p* = 0.186) FEV_1_ (%) (110.7 ± 13.3 × 108.5 ± 16.8) vs. (102.4 ± 17.2 × 103.3 ± 18.9) (*p* = 0.100) FEV_1_/FVC (%) (87.3 ± 3.1 × 85.4 ± 3.6) vs. (83.6 ± 5.3 × 84.6 ± 6.0) (*p* = 0.093)		IG (pre × post) vs. CG (pre × post) FVC (%) (80 ± 21 × 85 ± 21) vs. (79 ± 21 × 79 ± 23) (*p* = 0.70) FEV_1_ (%) (82 ± 24 × 89 ± 25) vs. (78 ± 25 × 78 ± 25) (*p* = 0.65) FEV_1_/FVC (%) (102 ± 12 × 104 ± 9) vs. (97 ± 17 × 97 ± 13) (*p* = 0.92)
**Author/Year**	**Maritescu et al./2024** [36]		**Sirydakis et al./2024** [37]	
Risk of bias	Low		Unclear	
Participants	IG (*n* = 30) vs. CG (*n* = 31)		IG (*n* = 21) vs. CG (*n* = 19)	
Intervention	IG—OPR supervised, in-person 5×/weeks (progressive aerobic and resistance training, and breathing exercises for 3 consecutive weeks). CG—OPR identical to that of IG, combined with 20 min of relaxation.		IG—OPR supervised with multicomponent 2×/week (aerobic and resistance exercises), with a total duration of 11 weeks, including 1 week for familiarization and 10 weeks of training. CG—recommendations on physical activity and sedentary behavior.	
Physical Performance (6MWT) (m)	IG (pre × post) vs. CG (pre × post) (332 ± 86.28 × 366 ± 81.97) vs. (347.25 ± 61.94 × 391.54 ± 56.81) (*p* < 0.001)			
EQ 5D QoL			IG (pre × post) vs. GC (pre × post) [(10.70 ± 0.77 × 8.83 ± 0.82)] vs. [(10.11 ± 0.81 × 11 ± 1.11)] (*p* < 0.001)	
PF	IG (pre × post) vs. CG (pre × post) in median/IQR FVC (%) [71.5 (67–81) × 76 (71–85)] vs. [72 (67–78) × 76 (69.25–83)] (*p* < 0.001) FEV_1_ (%) [78 (66–89) × 84.5 (73–94)] vs. [75 (64–86.75) × [79 (71.75–94.50)] (*p* < 0.001) FEV_1_/FVC (%) [84 (75–86) × 85.50 (76–88)] vs. [85 (76–88.75) × 85 (75–92.75)] (*p* = 0.001)			
Conclusion	Improvement in exercise performance, with or without relaxation as an additional intervention.		Improvement in QoL in the IG compared to CG.	

Abbreviations: IG—intervention group; CG—control group; OPR—outpatient pulmonary rehabilitation; WHO—World Health Organization; 6MWT—Six-Minute Walking Test; m—meters; 5 × SST—5-repetition Sit-to-Stand Test; vs.—versus; SF-12—Short Form-12; QoL/PD—quality of life; Qol/PD—QoL/physical domain; QoL/MD—quality of life/mental domain; QoL/EQ 5D—quality of life/EuroQol 5 dimensions; EQ/VAS—visual analog scale; EQI—EQ Index; PF—pulmonary function; FVC—forced vital capacity; FEV_1_—forced expiratory volume in one second; mMRC—modified Medical Research Council.

**Table 2:** Outcomes of Observational Cohort Studies (OCSs).

Author/Year	Daynes et al./2021 [38]	Hasenoehrl et al./2022 [39]	Mammi et al./2023 [40]	Moine et al./2024 [41]	Mujovic et al./2024 [42]
Risk of bias	Unclear	Low	Unclear	Unclear	Low
Participants	IG (*n* = 32)	IG (*n* = 28)	IG (*n* = 50)	IG (*n* = 47)	IG (*n* = 46)
Intervention	OPR supervised aerobic exercise (walking/treadmill), upper and lower limb strength training, 2×/week for 6 consecutive weeks.	OPR for subjects in both groups with resistance exercises, 2×/week for 8 consecutive weeks.	OPR individualized with progression to endurance training consisted of 10 individual 45-min sessions with physiotherapists 2×/week for 7 consecutive weeks. An additional 10 to 15 sessions were added.	OPR supervised with 6×/week (aerobic and strength exercises), for 4 consecutive weeks.	OPR individualized 5×/week (aerobic exercise) for 45 min for 6 consecutive weeks.
Physical Performance (6MWT) (m)		IG (pre × post) 640.70 ± 71.60 vs. 705.56 ± 75.60 (*p* < 0.001)		IG (pre × post) (*n* = 44) 519 ± 116 vs. 589 ± 124 (*p* < 0.001)	IG (pre × post) 506 ± 88.15 vs. 588 ± 128.15 (*p* < 0.001)
Physical Performance (30″SST)		IG (pre × post) 19.10 ± 6.0 vs. 27.01 ± 8.46 (*p* < 0.001)			
Physical Performance (1′SST)					IG (pre × post) in MD and IQR 24 (18–27.5) vs. 25 (22–31.5) (*p* < 0.01)
SF-12 QoL/PD				IG (pre × post) (*n* = 38) 33 ± 11 vs. 42 ± 9 (*p* < 0.001)	
SF-12 QoL/MD				IG (pre × post) (*n* = 38) 40 ± 10 vs. 50 ± 8 (*p* < 0.001)	
QoL (EQ 5D)	IG (pre × post) VAS 62 ± 18 vs. 70 ± 21 (*p* = 0.05)		IG (pre × post) VAS 60.23 ± 17.42 vs. 79.44 ± 16.48 (*p* < 0.001) EQI 0.65 ± 0.21 vs. 0.75 ± 0.20 (*p* < 0.001)		
Dyspnea (mMRC)				(*n* = 25) 1 (1–2) vs. 1 (1–1) (*p* < 0.01)	
Conclusion	Improving the QoL.	Improved exercise performance.	Improving the QoL.	Improved exercise performance, QoL/PD and QoL/MD.	Improved exercise performance.
**Author/Year**	**Ostrowska et al./2023** [43]	**Rzepka-Cholasińska et al./2024** [44]		**Silva-Lalucci et al./2024** [45]	**Tramonti et al./2024** [46]
Risk of bias	Unclear	Unclear		Unclear	Unclear
Participants	IG (*n* = 97)	IG (*n* = 97)		IG (*n* = 59)	IG (*n* = 18)
Intervention	OPR with aerobic and resistance exercises for 90 min each session, 3×/week for 6 consecutive weeks.	OPR with aerobic, strength and interval exercises with load progression, 3×/week for 6 consecutive weeks.		OPR with resistance and strength exercises, 2×/week for 16 consecutive weeks.	OPR with aerobic exercise, 3×/week for 4 consecutive weeks.
Physical Performance (6MWT) (m)	IG (pre × post) 335 ± 45 vs. 383.25 ± 51.50 (*p* < 0.0001)	IG (pre × post) 327.80 ± 82.88 vs. 383.96 ± 74.59 (*p* < 0.0001)		IG (pre × post) 524.30 ± 89.78 vs. 577.30 ± 90.96 (*p* < 0.0001)	IG (pre × post) 320 ± 184 vs. 395 ± 193 (*p* < 0.001)
Physical Performance (30″SST)	IG (pre × post) 13.0 ± 3.0 vs. 18.0 ± 3.5 (*p* < 0.0001)	IG (pre × post) 13.39 ± 4.32 vs. 17.10 ± 4.66 (*p* < 0.0001)		IG (pre × post) 16.06 ± 8.71 vs. 20.36 ± 8.73 (*p* < 0.0001)	
SF-36 QoL/PD					IG (pre × post) in % 57.5 [35] vs. 85 [19] (*p* = 0.001)
SF-36 QoL/MD					IG (pre × post) % 64 [21] vs. 68 [21] (*p* = 0.05)
Dyspnea (mMRC)	IG (pre × post) in median/IQR 2 (2–2) vs. 1 (0–2) (*p* < 0.0001)	IG (pre × post) 2.21 ± 0.52 vs. 1.00 ± 0.89 (*p* < 0.0001)			IG (pre × post) 3.0 ± 1.0 vs. 1.0 ± 1.0 (*p* < 0.0001)
Dyspnea (Borg)	IG (pre × post) 3.0 ± 1.0 vs. 2.0 ± 1.0 (*p* < 0.0001)	IG (pre × post) 3.65 ± 1.95 vs. 2.26 ± 1.44 (*p* < 0.0001)			
Conclusion	Improved exercise performance, reduced dyspnea.	Improved exercise performance and reduced dyspnea.		Improved exercise performance.	Improved exercise performance, QoL and reduced dyspnea.

Abbreviations: IG—intervention group; OPR—outpatient pulmonary rehabilitation; vs.—versus; h—hours; 6MWT—Six-Minute Walking Test; m—meters; 30″SST—30 s Sit-to-Stand Test; 1′SST—1 min Sit-to-Stand Test; SF-12—Short Form-12; QoL—quality of life; Qol/PD—QoL/physical domain; QoL/MD—QoL/mental domain; EQ 5D—quality of life/EuroQol 5 dimensions; EQ/VAS—visual analog scale; EQI—EQ Index; MD—median; IQR—interquartile range; PF—pulmonary function; FEV_1_—forced expiratory volume in one second; FVC—forced vital capacity; mMRC—modified Medical Research Council.

## Data Availability

The original contributions presented in this study are included in the article/Supplementary Material. Further inquiries can be directed to the corresponding author.

## Supplementary Materials

The following supporting information can be downloaded at: https://drive.google.com/drive/folders/1_1xbbHjZ0t6i0E_U4nUCje55EunGVb2i?usp=sharing, accessed on 6 April 2026. S1: Concept map; S2: Search strategy conducted; S3: Characteristics of the included studies; S4: List of excluded studies; Meta-analysis S5: Sensitivity analysis with SST30″; Meta-analysis S6: Effectiveness of OPR on QoL EQ 5D/VAS; Meta-analysis S7: Effectiveness of OPR on dyspnea by Borg; S8: Grade/certainty of evidence; S9: Funnel plot.

## Abbreviations

The following abbreviations are used in this manuscript:

LC	Long COVID
QoL	Quality of Life
PR	Pulmonary Rehabilitation
OPR	Outpatient Pulmonary Rehabilitation
FEC	Functional Exercise Capacity
PF	Pulmonary Function
RCTs	Randomized Clinical Trials
OCS	Observational Cohort Studies
PEDro	Physiotherapy Evidence Database
NHLBI	National Heart, Lung, and Blood Institute
MD	Mean
CI	Confidence Interval
I^2^	Heterogeneity
6MWT	Six-Minute Walk Test
SST	Sit-to-Stand Test
30″SST	30-Second Sit-to-Stand Test
1′SST	1 min Sit-to-Stand Test
5 × SST	5-repetition Sit-to-Stand Test
mMRC	modified Medical Research Council
D-12	Dyspnea 12
ATS/ERS	American Thoracic Society/European Respiratory Society
GLI	Global Lung Functional Initiative
FVC	Forced Vital Capacity
FEV_1_	Forced Expiratory Volume in One Second
ADL	Activities of Daily Living
CAPES	Coordination for the Improvement of Higher Education Personnel
MeSH	Medical Subject Headings
AMSTAR	Measurement Tool to Assess Systematic Reviews 2
EQ 5D 5L	EuroQoL 5 dimensions, 5 level
EQ/VAS	EuroQoL/Visual Analog Scale
EQI	EuroQoL/Index
SF-12	Short Form 12
SF-36	Short Form 36
QoL/PD	Physical Domain of SF-12
Qol/MD	Mental Domain of SF-12
MSCD	Minimally Significant Clinical Difference
GRADE	Grading of Recommendations Assessment, Development, and Evaluation
IG	Intervention Group
CG	Control Croup
WHO	World Health Organization
IQR	Interquartile Range
ITT	Intention-to-Treat
m	Meters
50th	50th percentile
DSQ	DePaul Symptom Questionnaire
CFS	Chronic Fatigue Syndrome
PEM	Post-Exertional Malaise
